# Common dietary flavonoids inhibit the growth of the intraerythrocytic malaria parasite

**DOI:** 10.1186/1756-0500-1-26

**Published:** 2008-06-18

**Authors:** Adele M Lehane, Kevin J Saliba

**Affiliations:** 1 School of Biochemistry and Molecular Biology, The Australian National University, Canberra, ACT 0200, Australia; 2 Medical School, The Australian National University, Canberra, ACT 0200, Australia

## Abstract

**Background:**

Flavonoids are abundant plant phenolic compounds. More than 6000 have been identified to date, and some have been shown to possess antiparasitic activity. Here we investigate the effects of a range of common dietary flavonoids on the growth of two strains of the human malaria parasite *Plasmodium falciparum*.

**Findings:**

A chloroquine-sensitive (3D7) and a chloroquine-resistant (7G8) strain of *P. falciparum *were tested for *in vitro *susceptibility to a range of individual dietary flavonoids and flavonoid combinations. Parasite susceptibility was measured in 96-well plates over 96 h using a previously described [^3^H]hypoxanthine incorporation assay. Of the eleven flavonoids tested, eight showed antiplasmodial activity against the 3D7 strain (with IC_50 _values between 11 and 66 μM), and all showed activity against the 7G8 strain (with IC_50 _values between 12 and 76 μM). The most active compound against both strains was luteolin, with IC_50 _values of 11 ± 1 μM and 12 ± 1 μM for 3D7 and 7G8, respectively. Luteolin was found to prevent the progression of parasite growth beyond the young trophozoite stage, and did not affect parasite susceptibility to the antimalarial drugs chloroquine or artemisinin. Combining low concentrations of flavonoids was found to produce an apparent additive antiplasmodial effect.

**Conclusion:**

Certain common dietary flavonoids inhibit the intraerythrocytic growth of the 3D7 and 7G8 strains of *P. falciparum*. Flavonoid combinations warrant further investigation as antiplasmodial agents.

## Findings

Flavonoids are ubiquitous plant phenolic compounds that consist of two aromatic rings linked by a three-carbon bridge (see Table 1 for structure). More than 6000 flavonoids have been identified to date, and their essential functions in plants include regulating growth and providing protection against pathogens (reviewed in [1]). They are currently attracting significant medical interest because of their antioxidant, antitumor, antiinflammatory, antimicrobial and antiviral activities. Flavonoids are present in fruits, vegetables, wine, tea and coffee. Estimates of the daily intake of flavonoids in developed countries are as high as 1–2 g [1].

**Table 1:** Flavonoid IC_50 _values for growth inhibition of *P. falciparum *

**Class**	**Major food sources [17]**	**Flavonoid**	**Chemical substitutions/modifications**	***In vitro *antiplasmodial activity (IC_50_, μM)^a^**		**LogD (pH 7.0)^b^**
					
				**3D7**	**7G8**	
Flavonols	Onions, kale, broccoli, apples, cherries, fennel, sorrel, berries, tea	Kaempferol	3, 5, 7, 4' – OH	33 ± 7	25 ± 2	2.40
		Myricetin	3, 5, 7, 3', 4', 5' – OH	40 ± 10	76 ± 23	2.06
		Quercetin	3, 5, 7, 3', 4' – OH	15 ± 5	14 ± 1	2.09
		Isoquercitrin	3-gluc, 5, 7, 3', 4' – OH	66 ± 10	66 ± 18	1.59
Flavones	Parsley, thyme, celery, sweet red pepper	Acacetin	5, 7 – OH 4' – OCH_3_	> 100	13 ± 2	2.77
		Apigenin	5, 7, 4' – OH	20 ± 3	13 ± 2	2.77
		Baicalein	5, 6, 7 – OH	32 ± 1	21 ± 6	3.18
		Chrysin	5, 7 – OH	18 ± 3	22 ± 4	2.79
		Luteolin	5, 7, 3', 4' – OH	11 ± 1	12 ± 1	2.62
Flavanones	Citrus fruits, prunes	Naringenin	5, 7, 4' – OH, single bond between 2 and 3	> 125	71 ± 10	2.17
Isoflavones	Legumes	Genistein	5, 7, 4' – OH, B ring attached to 3	> 50	29 ± 5	1.53
Chloroquine	NA	NA	NA	0.006 ± 0.0003	0.084 ± 0.026	ND

^a ^Values are the means (± S.E.M.) of results from three independent experiments, each performed in duplicate or triplicate. Parasite susceptibility was measured in 96-well plates over 96 h using the [^3^H]hypoxanthine incorporation assay [19]. ^b ^LogD (the octanol-water distribution coefficient) was predicted using PrologD (Pallas for Windows version 2.1, CompuDrug International Inc.). Good cell permeability requires a logD value of 2–5 [20]. NA, not applicable; ND, not determined.

A number of reports have demonstrated growth inhibitory effects of flavonoids, particularly of the flavonol quercetin and of the flavone luteolin, on the protozoan parasite genera *Toxoplasma *[2], *Trypanosoma *[3] and *Leishmania *[4-6]. The majority of studies involving flavonoids and malaria describe the antiplasmodial activity-guided fractionation of plants (including species used in traditional medicine). Flavonoids (usually along with other compounds) have been identified in the antiplasmodial fractions of many plants, and in some cases have been shown to possess antiplasmodial activity when isolated [4,7-12]. Despite this, until very recently only the structurally distinct chalcone subclass of flavonoids attracted much further investigation.

In this study, the flavanone naringenin, the isoflavone genistein, and a range of flavonols (kaempferol, myricetin, quercetin and isoquercitrin) and flavones (acacetin, apigenin, baicalein, chrysin and luteolin) were tested against a chloroquine-sensitive (3D7) and a chloroquine-resistant (7G8) strain of *Plasmodium falciparum*. The flavonoids were purchased from Sigma-Aldrich (Australia), and purities were ≥ 95% except for isoquercitrin which was ≥ 90%. A recent study demonstrated the *in vitro *antiplasmodial activities of a number of these compounds [13]; we confirm and extend these findings with different *P. falciparum *strains.

Of the eleven flavonoids tested, eight showed antiplasmodial activity against the 3D7 strain, with mean IC_50 _values in the range 11–66 μM (Table 1). The remaining three (acacetin, naringenin and genistein) did not achieve 50% parasite killing at the highest concentrations tested (which varied according to solubility in aqueous media; DMSO concentrations were kept ≤ 0.1% to prevent any effect on parasite growth). All the flavonoids tested showed measurable activity against the chloroquine-resistant 7G8 strain, with IC_50 _values between 12 and 76 μM. The most active compound in both strains was luteolin, with IC_50 _values of 11 ± 1 μM and 12 ± 1 μM for 3D7 and 7G8, respectively (Table 1).

Marked differences in the susceptibilities of the two strains to some flavonoids (most notably to acacetin) were observed in this study and in that of Tasdemir *et al*. [13]. In both studies, the chloroquine-resistant strain was generally more susceptible to growth inhibition. Whether enhanced sensitivity to some flavonoids relates to the chloroquine resistance phenotype or other difference(s) in genetic background is not known. These strain differences make structure-activity determinations difficult. Furthermore, potential differences in cell permeability should also be considered. The higher antiplasmodial activity of quercetin (logD 2.09) compared to the quercetin glucoside isoquercitrin (logD 1.59) (P = 0.002, paired t-test) could be a result of better cell permeability. Similarly, the flavanone naringenin (logD 2.17) differs from apigenin (logD 2.77) only in the absence of a double bond in the C ring but is much less active (P = 0.02, paired t-test for 7G8). Also, the isoflavone genistein (logD 1.53) differs from apigenin (logD 2.77) only in that the B ring is attached to carbon 3 of the C ring rather than carbon 2, and is less active against both strains (P = 0.03, paired t-test for 7G8). However, the testing of a number of additional flavonoids would be required to conclude that differences in activities are a result of different cell permeabilities.

The effect of a number of the flavonoids tested here on the viability of two normal (non-cancerous) human cell lines has been reported [14]. In the cases of apigenin, kaempferol, luteolin and quercetin, the IC_50 _values against both strains of *P. falciparum *are at least 4-fold lower than the IC_50 _values reported against both human cell lines. Naringenin has relatively low activity against *P. falciparum *(Table 1) and human cells [14]. In addition, Mamani-Matsuda et al. [3] investigated quercetin toxicity towards haemopoietic cells and found that the IC_50 _value was greater than 100 μM (> 6-fold higher than the IC_50 _against *P. falciparum *reported here; Table 1).

**Figure 1:**
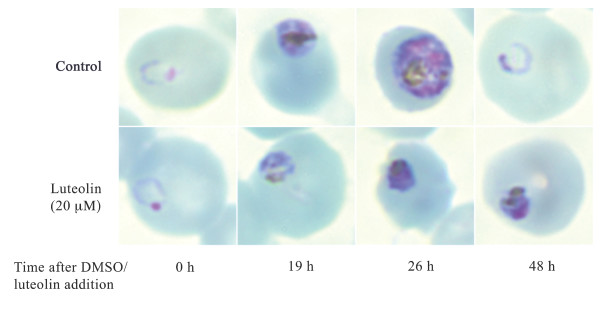
**The effect of luteolin on the intraerythrocytic growth of *P. falciparum***. Giemsa-stained smears were prepared at 0, 19, 26 and 48 h after the addition of luteolin (20 μM) dissolved in DMSO or an equal volume of DMSO (control; 0.025% DMSO) to 7G8-infected erythrocytes (1% parasitemia, 2% hematocrit). While control parasites developed into mature trophozoites (26 h) that subsequently gave rise to daughter parasites (48 h), the growth of luteolin-treated parasites was arrested at the young trophozoite stage. Luteolin-treated parasites did not give rise to daughter parasites, resulting in a reduced parasitemia compared to the control at the 48 h time point.

To investigate which stage of the parasite's intraerythrocytic lifecycle is inhibited by flavonoids, we prepared Giemsa-stained 7G8-infected erythrocyte smears at various time points following the addition of 20 μM luteolin (~2 × IC_50_). Figure 1 reveals that luteolin does not inhibit the ring stage of the parasite but prevents the progression of parasite growth beyond the young trophozoite stage. The parasites are therefore unable to complete a full intraerythrocytic cycle. An arrest in cell cycle progression has also been observed for *Leishmania donovani *promastigotes treated with luteolin or quercetin [5]. Furthermore, quercetin has been shown to inhibit bradyzoite development in *Toxoplasma gondii *[2]. Further studies are required to elucidate the mechanism by which flavonoids arrest parasite growth.

There have been several reports of flavonoids showing synergistic or antagonistic activities with various antiparasitic drugs [10,15,16]. We therefore tested the effect of luteolin (at concentrations of 2.5 μM, 5 μM, 10 μM and 20 μM) on the susceptibility of 7G8 to two important antimalarial drugs, chloroquine and artemisinin. Luteolin did not significantly affect the IC_50 _values of either drug (not shown). Therefore, luteolin should not antagonise the activities of chloroquine or artemisinin if used in combination therapy.

**Figure 2:**
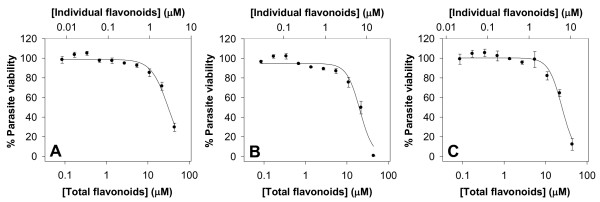
**The effects of flavonoid combinations on the proliferation of the 7G8 strain of *P. falciparum***. Equimolar amounts of (A) the eleven flavonoids tested in this study, (B) apigenin, luteolin and quercetin, and (C) apigenin, luteolin, naringenin and quercetin, were combined. The data are averaged from three independent experiments, each performed in duplicate or triplicate. Error bars represent S.E.M.

The finding that common dietary compounds display antiplasmodial activity raises the intriguing question of whether a plant-based diet could play a role in malaria prevention or lessen disease severity. To investigate whether combinations of dietary flavonoids could display an additive effect, we combined equimolar amounts of the eleven flavonoids tested in this study such that the highest concentration of each was not toxic to the parasite (4 μM). This resulted in 50% growth inhibition against 7G8 at a total flavonoid concentration of 31 ± 3 μM (Figure 2A), a value similar to the mean IC_50 _value for the eleven flavonoids (33 μM). At this total flavonoid concentration, each individual flavonoid is present at a concentration of only 2.8 μM. We then combined equimolar amounts of three of the most active compounds, luteolin, quercetin and apigenin, such that the highest total flavonoid concentration was 44 μM (i.e. the same maximum total flavonoid concentration used when 11 flavonoids were combined). The combination of these three flavonoids gave an IC_50 _value of 21 ± 2 μM (Figure 2B), somewhat higher than their mean IC_50 _value (13 μM). However, at this total flavonoid concentration each individual flavonoid is only present at a concentration of 7 μM, which is lower than individual IC_50 _values. Adding a less active flavonoid (naringenin) to this combination, while maintaining the same maximum combined flavonoid concentration of 44 μM (i.e. each individual flavonoid was now present at a maximum concentration of 11 μM), increased the IC_50 _value close to the new mean value (combination IC_50 _= 24 ± 2 μM, average IC_50 _= 28 μM; Figure 2C). While the data are consistent with the flavonoids having an additive antiplasmodial effect, the possibility that there is a combination of synergistic and antagonistic effects between some of the individual flavonoids in these combinations that results in an apparent additive effect cannot be excluded.

The concentrations of diet-derived individual flavonoids in plasma are unlikely to reach those required for complete parasite killing. The extent to which flavonoids are absorbed from the gastrointestinal tract depends on factors such as the flavonoid itself and the dietary source [1,17,18]. Another important issue is the metabolism of flavonoids *in vivo *to form potentially less active conjugates [18]. Thus, it will be important to investigate the activities of the main conjugates of promising antiplasmodial flavonoids. Nevertheless, our study raises the possibility of using clinically relevant, non-toxic concentrations of flavonoid combinations as antiplasmodial agents.

## Competing interests

The authors declare that they have no competing interests.

## Authors' contributions

AML participated in the design of the study, performed the experiments and data analysis and drafted the manuscript. KJS participated in the design of the study and helped to draft the manuscript. Both authors read and approved the final manuscript.
